# Virtuous and Vicious Virtual Water Trade with Application to Italy

**DOI:** 10.1371/journal.pone.0093084

**Published:** 2014-04-11

**Authors:** Julia Anna Winter, Paola Allamano, Pierluigi Claps

**Affiliations:** Department of Environment, Land and Infrastructure Engineering, Politecnico di Torino, Torino, Italy; Universidad Veracruzana, Mexico

## Abstract

The current trade of agricultural goods, with connections involving all continents, entails for global exchanges of “virtual” water, i.e. water used in the production process of alimentary products, but not contained within. Each trade link translates into a corresponding virtual water trade, allowing quantification of import and export fluxes of virtual water.

The assessment of the virtual water import for a given nation, compared to the national consumption, could give an approximate idea of the country's reliance on external resources from the food and the water resources point of view. A descriptive approach to the understanding of a nation's degree of dependency from overseas food and water resources is first proposed, and indices of water trade virtuosity, as opposed to inefficiency, are devised. Such indices are based on the concepts of self-sufficiency and relative export, computed systematically on all products from the FAOSTAT database, taking Italy as the first case study. Analysis of time series of the self-sufficiency and relative export can demonstrate effects of market tendencies and influence water-related policies at the international level.

The goal of this approach is highlighting incongruent terms in the virtual water balances by the viewpoint of single products. Specific products, which are here referred to as “swap products”, are in fact identified as those that lead to inefficiencies in the virtual water balance due to their contemporaneously high import and export. The inefficiencies due to the exchanges of the same products between two nations are calculated in terms of virtual water volumes. Furthermore, the cases of swap products are investigated by computing two further indexes denoting the ratio of virtual water exchanged in the swap and the ratio of the economic values of the swapped products. The analysis of these figures can help examine the reasons behind the swap phenomenon in trade.

## Introduction

The virtual water content of a product is the volume of water used to produce it, measured at the place where it is actually grown or manufactured. The adjective “virtual” refers to the fact that most of the water used in the production is ultimately not contained within the product. The real water content of products is generally negligible compared with their virtual water content [1].

The virtual water content of a given product varies greatly from place to place, depending mainly on the climate and on the technology adopted for irrigation and farming [2]. The variability of virtual water contents among different products and different locations has led to a diversification of case studies and research topics. Studies have been conducted at a global scale (e.g. [3], [4], [5]) but also in depth on single countries or areas (e.g. [6], [7], [8]) or on single products (e.g. [9], [10], [11]).

The global trade of all goods can also be translated into a corresponding network of virtual water trade, allowing quantification of the import and export fluxes of virtual water (e.g. [12], [13], [14]). Nations have been classified as net importers or exporters of virtual water according to the balance of trade fluxes entering and leaving the country (e.g. [15]).

It is therefore necessary to address the problems concerning water resources, such as water scarcity, water excess and deterioration of quality, not only at the agricultural district level and at the river basin level, but also at the nation and, ultimately, at a global level [15].

A nation can be considered “wise” when it exports products that are obtained from abundantly available resources within the country and imports products that are produced with resources that are scarcely available within the country. One of these resources could clearly be water; a water-scarce country might thus aim at behaving “wisely” by importing products that require a lot of water in their production and exporting products or services that require less water. However, this “virtuous” kind of trade does not (always) correspond to what trade figures show when examined on a long term and at the nation level, most likely because other factors, such as profit for instance, have a stronger influence on trade patterns.

To better examine this concept, it is important to determine the significance of the virtual water import for a given nation compared to what is nationally consumed; this could give an approximate idea of the country's reliance and dependency on external resources. The resources taken into consideration for this study are water resources destined for food production. Using the virtual water concept, a descriptive approach of the degree of dependency of a nation from overseas water resources is expanded in the next section in which indices are created, that could suggest a degree of trade “virtuosity” or “inefficiency”. This requires establishing virtual water balances between a country and the rest of the world, in terms of exchanges of crops and livestock.

The immediate goal of this approach is to highlight incongruent terms in the virtual water balances, at a nation's scale and by the viewpoint of single products. In the subsequent sections, specific incongruences emerging in the trade of single products are highlighted to allow a clearer connection between economic driving forces and water resources. Italy is the nation considered in all examples shown in the paper, because it is among the leading OECD (Organisation for Economic Co-operation and Development) countries in the list of the greatest virtual water importers [8].

## Materials and Methods

### Descriptive Analysis of Production and Overall Trade

Data necessary for the investigations on production and trade presented in this paper are obtained from the database FAOSTAT (www.faostat.fao.org), that provides time-series of data relating to food and agriculture for some 200 countries. For this analysis primary and processed crops and primary and processed livestock are taken into consideration: 397 items in total for Italy - from year 1961 to year 2009. Exchanges are considered in terms of quantities imported (I), exported (E) and produced (P), in tonnes. Production data are paired with data on import and export and the balance term ‘Consumption’ (C) is defined as C = P−E+I (considering constant storage stocks).

In order to have an overview of the overall trade of the country of interest, two ratios are computed for each product throughout the available time span:

- the ratio between ‘Import’ and ‘Consumption’ (I/C), that shows the countries' dependency on overseas products; this term, named DP after *dependency*, indicates how much of the national request for crops or livestock is satisfied by import and not by internal production (the closer DP is to 0 the more is the commodity supply provided by the country itself);
- the ratio between ‘Export’ and ‘Production’ (E/P), named ER for *export ratio*, that shows the relative export attitude of the country; the index ER explains how much of the internal production is destined to be exported.

Items which have a high DP are items whose national demand is not satisfied by internal production; those which have a high ER are items whose production is destined mostly to be exported instead of being nationally consumed. When combining these two metrics, products can be found which show both a high DP and a high ER. These are products which behave very particularly: whilst there is a significant import, seemingly because of an impossibility of self sufficiency of the nation, there is for the same commodity a strong export of its production. Acknowledging that further internal processing of products is impossible to track, and assuming that the country does not function as a mere transit, i.e. imports are not consumed nationally but further exported, this feature configures a “vicious” kind of trade. The interest in computing DP and ER systematically on all products and on all the available years is in demonstrating possible “vicious” and incongruent trade figures for a given nation, at least in terms of virtual water budget.

**Figure 1:**
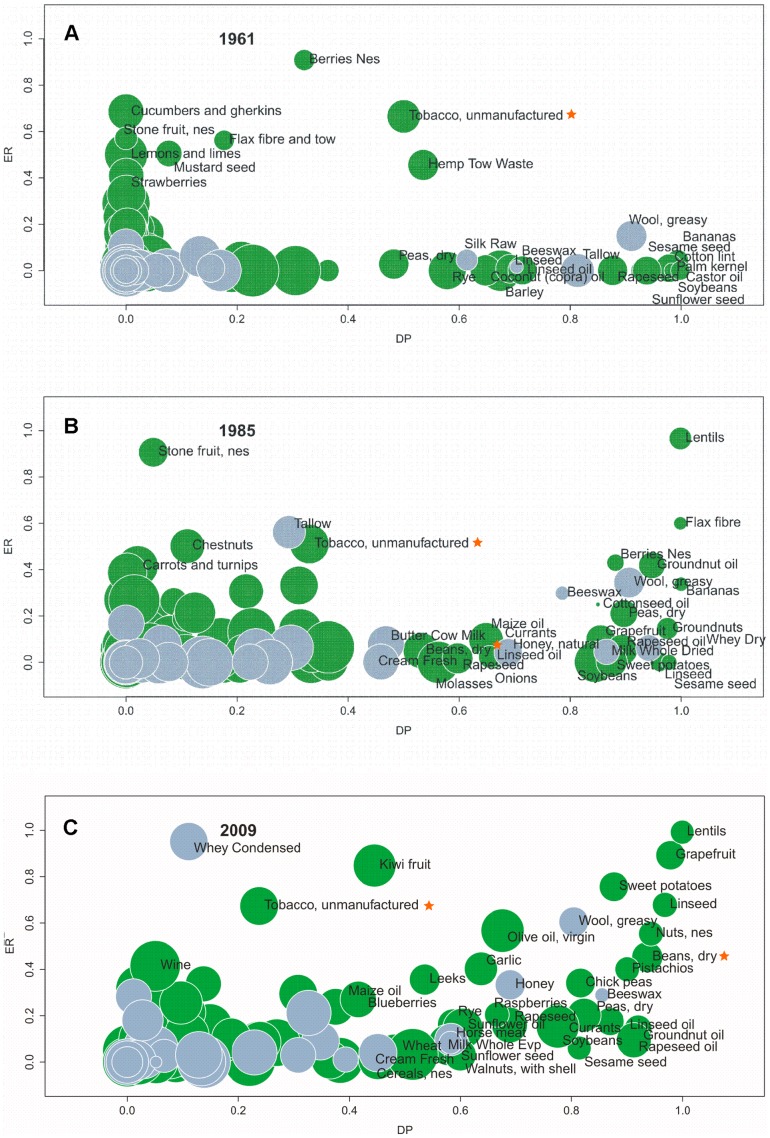
ER-DP diagram for Italy for years 1961, 1985 and 2009. The indices of dependency DP and export ratio ER are computed over all products available and are visualised on a bubble graph where the horizontal axis shows DP and the vertical axis ER. The production amount is reflected in the size of the bubbles, that also varies among the years. The radius of the bubble is assumed to scale with production amounts according to a logarithmic law, i.e. when the radius doubles, a factor of 10 is applied. Logarithmic scaling allows to appreciate figures for the smallest productions. Everything is then scaled by the numeric amount of the highest production (for years 1961, 1985, 2009 respectively: Grapes 8,5E+06 tonnes, Grapes 9,6E+06 tonnes, Grapes 8,2E+06 tonnes). The colour green indicates primary and processed crops whereas light blue stands for primary and processed livestock. The orange stars indicate the position of beans and tobacco in reference to Figure 2.

Considering several products one may want to visualize the above indices on a bubble graph where the horizontal axis shows the dependency DP and the vertical axis the export ratio ER (Fig. 1). The production amount is reflected in the size of the bubbles, that also varies among the years. The radius of the bubble is assumed to scale with production amounts according to a logarithmic growth, i.e. when the radius doubles, a factor of 10 is applied. Logarithmic scaling allows to appreciate figures for the smallest productions. Everything is then scaled by the numeric amount of the highest production (that is reported in the caption of the figure).

The main information content of fig. 1 is the position of a commodity according to the quadrant where the two indices fall. The items located in the lower left quadrant are those which have very simple behaviour: they are produced locally mainly for the local consumption, keeping export and import to the minimum. This otherwise means that the production of such items seems to be calibrated on the request of the population and does not exceed it. The commodities located towards the upper right quadrant of the (DP, ER) diagram, instead, have at the same time high import and high export amounts compared respectively to consumption and production. These positions hence exemplify the incongruent type of trade discussed above. Figure 1 shows the ER-DP representation for the totality of the products analysed for Italy in three different years (1961, 1985 and 2009) which were taken as indicative of the complete time series. The colour green indicates primary and processed crops whereas light blue stands for primary and processed livestock. The products located in the upper right quadrant have evidently increased in number from 1961 to the year 2009.

**Figure 2:**
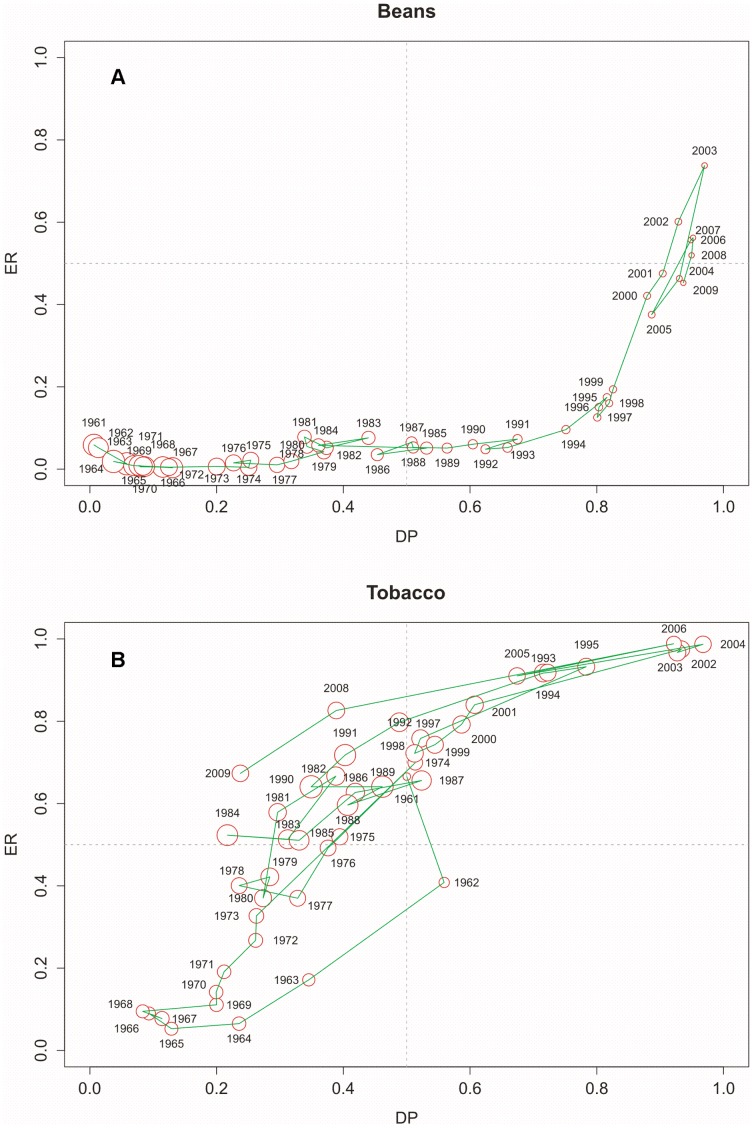
ER-DP diagram for Italy of Beans and Tobacco 1961–2009. Visualisation of the time trend of a single product on the ER-DP diagram. The examples chosen for this time-series representation are Beans and Tobacco, whose location in the ER-DP diagram is highlighted by an orange star in Figure 1 (beans in 1961 were below the threshold for labelling). For both of these crops, DP and ER data have been plotted versus time. It is evident that for these commodities there has been an increase in time in both DP and ER. The radius of the bubble is again proportional to the production, but is not scaled in a logarithmic way; the numeric amount of the highest production is 2,1E+05 tonnes in case of Beans in 1964 and 2,1E+05 tonnes for Tobacco in 1990.

A different way of examining information on production and trade is to visualize the time trend of a single product on the ER-DP diagram. The examples chosen for this time-series representation are Beans and Tobacco, whose location in the ER-DP diagram is highlighted by an orange star and that -among the products with high DP, high ER or both- presented the most significant trends. DP and ER data have been plotted versus time in figure 2, that shows for these commodities an increase in time in both DP and ER. The radius of the bubble is again proportional to the production but is not scaled by logarithm; the numeric amount of the highest production is reported in the caption of the figure. Economic considerations are likely to justify this peculiar evolution, as will be discussed hereinafter.

## Results and Discussion

### Virtual Water Inefficiency Assessment: The Swap Phenomenon

The previously shown behaviour for which a commodity is at the same time highly imported and highly exported will be referred to as the “swap” attitude. Between two trading countries a product can therefore be considered a swap product when being “unreasonably” exchanged both ways.

In order for a country to quantify the actual swap, information is necessary regarding import and export considering all fluxes entering (leaving) Country #1 from (to) the individual country of origin (destination). Such information can be obtained from a further section of the FAO database (i.e. the FAO Statistics TradeSTAT). A specific evaluation of the entity and evolution of this attitude is conducted on Italy as an initial case study.

The quantification of swap for the various products is done by comparing, year by year, the products' amounts traded by Italy with each different country: the minimum between import and export of each trade connection is selected as the quantity of product which is unreasonably exchanged, as expressed in the following equation:
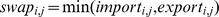
where i indicates the traded commodity and j the country with which Italy is trading it. This is because a product should (ideally) only be imported or exported. When both terms are different from zero the smallest of the two can be considered “unnecessary”. In other words, if the minimum is zero, there is an understandable dependency of one of the two countries (Italy in the specific case) on the other country. If this minimum is different from zero there is overlapping between I and E and this overlap is the swap amount.

**Figure 3:**
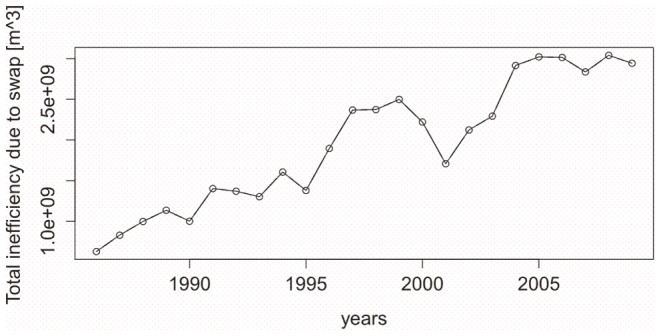
Total inefficiency assessment in terms of volume of virtual water traded through swap amounts. Total inefficiency assessment in terms of volume of virtual water traded through swap amounts, computed for Italy. The total (in tonnes) of the swap exchanges for each commodity is calculated by adding up all contributions from each trading country. The translation of these data into quantities of virtual water is subsequently done by multiplying the total swap amounts by the virtual water content per ton of each commodity. This turns the product amounts into volumes of virtual water inefficiently exchanged for each commodity; for the assessment of the annual totals it is simply sufficient to add up all products contributions. This calculation is conducted for each year from 1986 to 2009 over all products reported by FAO. Results are presented as the dotted time series.

The total (in tonnes) of the swap exchanges for each commodity is calculated by adding up all contributions from each trading country. The translation of these data into quantities of virtual water is subsequently done by multiplying the total swap amounts by the virtual water content per ton of each commodity, assessed for Italy [3], [8], [16]. This turns the product amounts into volumes of virtual water, in m^3^, inefficiently exchanged for each commodity; for the assessment of the annual totals it is simply sufficient to add up all products contributions. This calculation is conducted for each year from 1986 to 2009 over all products reported by FAO. Results are presented as a dotted time series in Figure 3. A significant increasing trend is observed for the total volumes of virtual water traded through swap amounts at the nation's level on all trade connection. In order to understand the entity of the swap phenomenon a significant comparison with the total net flux can be done for the Italian case study: in 2009 the average net import of virtual water is 50E+09 m^3^ [8] while the swap entails for 3E+09 m^3^.

In the supporting information, Figure S1 and Figure S2, shown are synthetic representations of the virtual water carried from unnecessary imports, per commodity, for year 2009. The products with a null result of swap amount were omitted from this already “dense” representation. Of the totality of 397 items in year 2009, 365 undergo the swap phenomenon, with differences in quantity and in the number of countries involved. Of 365 items only for 162 was the virtual water content (m^3^/ton) available and therefore the computation of the inefficiency in terms of traded virtual water possible. A logarithmic x axis is used to cover the whole range of trading amounts. For the sake of a clear representation the totality of the 162 products is divided into two categories, called ‘crop’ and ‘livestock’. From this moment onwards the pool of products which will be taken into consideration for further analysis consists in the 162 commodities for which information on water content was obtainable.

While studying the possible explanations behind these vicious exchanges, two main considerations emerge regarding the phenomenon.

Firstly, the existence of swap entails that a country may work as a transit, i.e. products are passing through the country to reach their final destination. A method to pinpoint the manifestations of this phenomenon in the trade of a single country is to detect the products with a higher import than consumption (DP>1). This behaviour, corresponding also to higher export than production (ER>1), indicates a transportation of the product in question through the country, which may just act as a sorter. The values of the ratios ER and DP previously computed for all products and all years were scanned, allowing to identify a few products, such as Cotton lint, Mustard seed, Bananas and Flax fibre & tow, which show such a behaviour in Italy.

Secondly, many of the trade links between countries from different hemispheres are justified by the seasonality of some cultivations and allow to obtain a year-round availability of the commodities in question. The values obtained from FAO refer to the whole year which makes these trade links not immediately detectable. A more sensible analysis is needed in order to avoid considering swap for what is possibly an import or export of seasonal relevance; this could be further attempted by considering only the European countries, with which Italy shares climate and seasonality in crop production. The relevance of this subset finds support in Tamea et al. (8) from which it emerges that 60% of the total import of virtual water for Italy in 2010 comes from Europe and 72% of the export of virtual water from Italy in the same year is destined to Europe. European countries as a whole therefore represent the main trading partners for Italy. In particular, France is the major supplier of virtual water and one of the greatest importer of Italian goods, second only to Germany.

### Swap Analysis

The analyses reported so far provide figures that raise questions about the sustainability of swap trade, as reasons behind the anomalies of swap products could be numerous. The attempt made is to investigate each of the previous trade connections which present a swap from an economic point of view and in terms of water resources efficiency. To this purpose, two ratios are introduced which are firstly computed for all trading links and, at a later stage, examined in depth for those products presenting the highest quotes of swap, as they result from the previous section.

The first indicator, referred to as Virtual Water Volume Ratio, is a ratio between the virtual water content (m^3^/ton) of an item leaving Italy as export for another country, and the virtual water content (m^3^/ton) of that item entering Italy from that same country. The ratio is computed for all items and all countries, generating a matrix of Virtual Water Volume Ratios, with a value for each trade connection. A “spatial” weighted mean is calculated for each product by using the import quantities from each country as weights, resulting into a single average value for each commodity. A Virtual Water Volume Ratio higher than 1 indicates a commodity which averagely requires more water for its production in Italy than in all other countries from which Italy is importing it. Table S1 reports the complete list of items considered in the computation. Of the 162 commodities for which the calculation was conducted, 93 present a Virtual Water Volume Ratio higher than 1.

The second indicator is the ratio between the economic unit value (1000USD/ton) of a certain commodity leaving Italy towards another country and the economic unit value (1000USD/ton) of the same commodity entering Italy from that same country. Information on such figures is obtained from the previously cited FAO Statistics TradeSTAT database. This ratio can justify the swap of products, since it is singularly calculated on each trading link between Italy and every single other nation on products which are being traded both ways. It will be referred to as Value Ratio and allows to pinpoint trade exchanges which are economically convenient in terms of unit value. As was done for the Virtual Water Volume Ratio, a matrix of Value Ratios is obtained when investigating each trading link and the weighted mean for each product is computed by using the swap (in tonnes) of each trade connection as weight. Table S2 presents the complete list of Value Ratio for the pool of commodities considered. The calculated Value Ratio is higher than 1 in 123 cases, meaning that Italian products are on average more valuable than the foreign product with which they are swapped. Goods exported from Italy thus present an added-value that could justify the swap.

**Figure 4:**
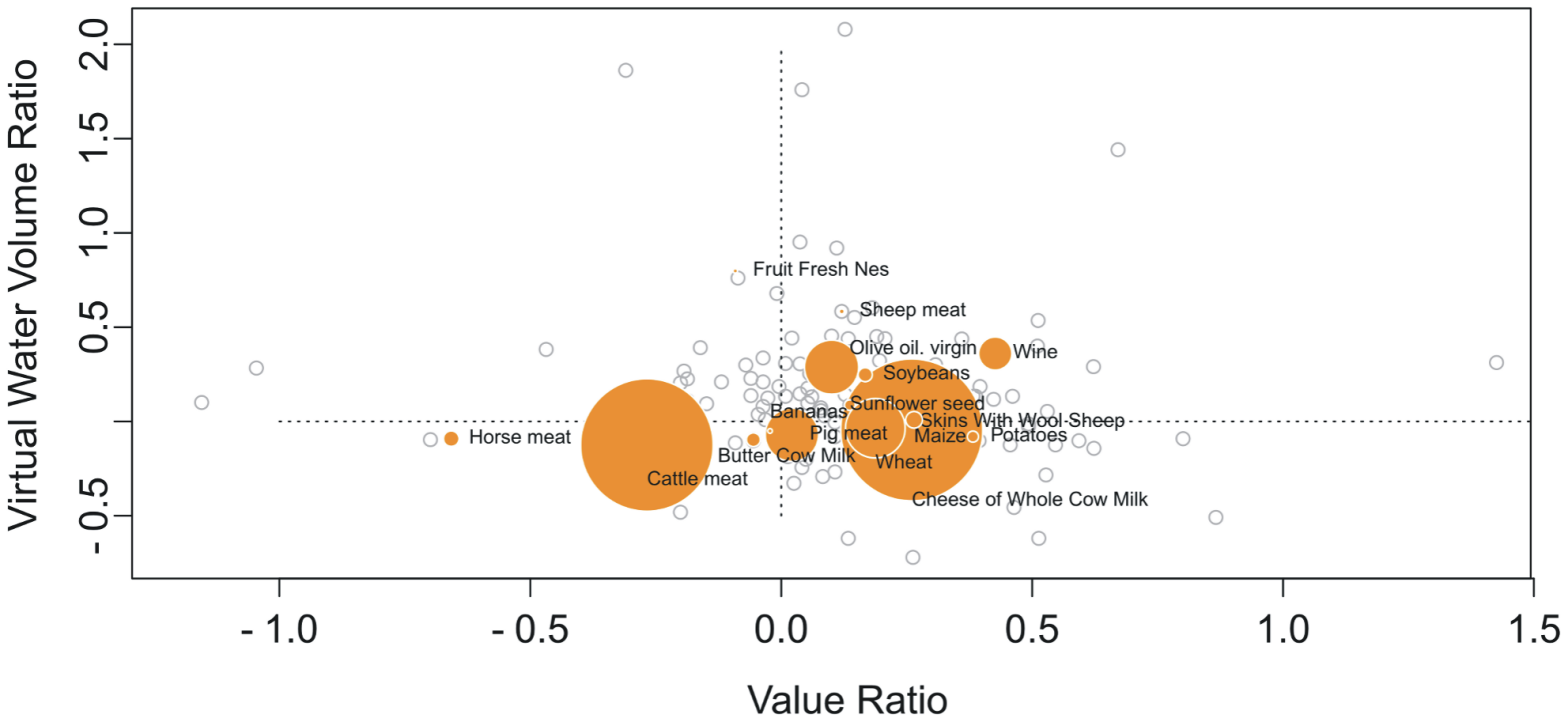
Value Ratio – Virtual Water Volume Ratio diagram for Italy in 2009. The indices of Value Ratio and Virtual Water Volume Ratio are computed over all products available and are presented as a scatter plot. Among the commodities produced in Italy in 2009 those with a swap higher than 10∧7 m^3^ are represented by bubbles whose dimension is given by the swap amount. The radius of the bubbles is scaled (non logarithmically) from the highest figure which is 3.2E+08 m^3^ of Cheese of Whole Cow Milk.

Figure 4 presents the totality of commodities taken into consideration (162 items) plotted on a bubble graph where the horizontal axis shows the Value Ratio and the vertical axis the Virtual Water Volume Ratio (both axis are in logarithmic scale). Among those commodities produced in Italy in year 2009, those with a swap higher than 10E+07 m^3^ are represented as orange bubbles, while the swap amount is reflected in the size of the bubbles (the radius is scaled according to the swap amount). It can be seen that, with the notable exception of cattle meat, the most important commodities from the point of view of swap fall in the right part of the graph, indicating a profit. The opposite behaviour could be ascribed to other drivers which determine the existence of some trade links, as for instance the ones related to the quality of imported products or to the customs and habits among neighbouring countries. These categories of drivers that influence the global food trade logics are extremely difficult to map and to quantify and are not investigated in this paper.

Data presented in tables 1 and 2 show the 20 most impacting items from the point of view respectively of m^3^ of virtual water inefficiently swapped (Table 1) and of what is referred to as net flux (8) (difference between the m^3^ of virtual water imported and m^3^ of virtual water exported, Table 2). The reported figures are coupled with the value and ranking (in brackets) of the two previous ratios, the Virtual Water Ratio and the Value Ratio. It can be noticed that many of the products which present a high swap, have at the same time a high value of net flux, such as wheat, meat-cattle boneless(beef and veal), maize, horse meat, soybeans. For a product presenting a high value of net flux, the country is strongly relying on import and consequently on the import of virtual water in order to satisfy the national demand of such commodity. A swap in such case would be most unwelcome and unreasonable, even if small in percentage. This is the case of wheat. Wheat, in fact, represents a highly traded crop (net flux of 8.3E+09 m^3^ of virtual water) and at the same time a highly swapped crop (swap of 1.3E+08 m^3^). By analysing the Virtual Water Volume Ratio one realises that, on average, the virtual water content of imported wheat is higher than the virtual water content of Italian wheat (the Italian contains 0.63 times the water averagely contained by foreign wheat). However, according to the Value Ratio, the wheat imported is economically less valuable than the Italian one (the Italian wheat has an economic unit value which is 1.54 times the average incoming wheat value). One could conclude, also in this case, that Italy swaps wheat in order to profit from it.

**Table 1:** The 20 most swapped products.

FAO code	Item	Swap (m3)	Virtual Water Volume Ratio	Value Ratio
870	Meat-CattleBoneless(Beef&Veal)	4.9E+08	0.79 (129)	0.88 (136)
901	Cheese of Whole Cow Milk	3.2E+08	0.90 (106)	1.82 (44)
867	Cattle meat	3.0E+08	0.75 (134)	0.54 (154)
15	Wheat	1.3E+08	0.63 (105)	1.54 (66)
261	Olive oil, virgin	1.2E+08	1.94 (34)	1.26 (93)
1035	Pig meat	1.2E+08	0.85 (112)	1.05 (117)
1039	Bacon and Ham	9.6E+07	0.81 (117)	2.21 (30)
564	Wine	7.4E+07	2.29 (24)	2.67 (19)
920	Hides Wet Salted Cattle	7.4E+07	0.81 (120)	0.95 (128)
921	Hidesdry S.Cattle	7.0E+07	0.77 (132)	0.81 (142)
268	Sunflower oil	5.7E+07	1.35 (64)	1.15 (102)
1232	Food Prep Nes	5.6E+07	2.51 (20)	1.44 (74)
898	Milk Skimmed Dry	4.7E+07	0.84 (113)	0.42 (157)
56	Maize	3.7E+07	1.02 (90)	1.84 (42)
16	Flour of Wheat	3.6E+07	0.63 (142)	1.12 (108)
1097	Horse meat	3.5E+07	0.81 (121)	0.22 (159)
1042	Prep of Pig Meat	3.3E+07	0.82 (116)	2.31 (27)
236	Soybeans	3.2E+07	1.77 (40)	1.47 (72)
886	Butter Cow Milk	3.1E+07	0.80 (124)	0.88 (135)
18	Macaroni	3.0E+07	0.93 (102)	0.97 (126)

The 20 most swapped products in terms of virtual water inefficiently traded, with relative Virtual Water Volume Ratio and Value Ratio.

**Table 2:** The 20 products with the highest net flux.

FAO code	Item	Net flux (m3)	Virtual Water Volume Ratio	Value Ratio
15	Wheat	8.3E+09	0.63 (105)	1.54 (66)
236	Soybeans	3.1E+09	1.77 (40)	1.47 (72)
238	Cake of Soybeans	2.2E+09	2.02 (29)	1.09 (112)
882	Cow milk, whole, fresh	1.9E+09	4.01 (10)	1.52 (68)
867	Cattle meat	1.7E+09	0.75 (134)	0.54 (154)
56	Maize	1.4E+09	1.02 (90)	1.84 (42)
268	Sunflower oil	1.4E+09	1.35 (64)	1.15 (102)
870	Meat-CattleBoneless(Beef&Veal)	1.3E+09	0.79 (129)	0.88 (136)
271	Rapeseed oil	1.3E+09	0.55 (148)	1.78 (48)
261	Olive oil, virgin	1.1E+09	1.94 (34)	1.26 (93)
1035	Pig meat	1.1E+09	0.85 (112)	1.05 (117)
267	Sunflower seed	9.4E+08	1.22 (75)	1.37 (81)
1097	Horse meat	8.9E+08	0.81 (121)	0.22 (159)
920	Hides Wet Salted Cattle	8.7E+08	0.81 (120)	0.95 (128)
901	Cheese of Whole Cow Milk	8.2E+08	0.90 (106)	1.82 (44)
269	Sunflower Cake	6.0E+08	1.47 (55)	1.59 (61)
44	Barley	5.0E+08	0.88 (109)	1.67 (56)
231	Almonds Shelled	4.2E+08	1.46 (56)	1.45 (73)
237	Soybean oil	3.7E+08	1.86 (36)	1.35 (84)
888	Milk Skm of Cows	3.4E+08	0.81 (122)	6.32 (3)

The highest 20 products from the point of view of the net flux (difference between the virtual water totally imported and exported), with relative Virtual Water Volume Ratio and Value Ratio.

## Conclusions

Relations between food production and the related water consumption are investigated by examining the nature and the evolution of bilateral trade between countries, with particular reference to the virtual water efficiency or inefficiency connected to production and trade. The research presented here relates not so much to the marginal production efficiency, which will depend on the characteristics of irrigation techniques or to the variations of productivity according to climate conditions; it rather considers the influence of commercial interests on the global trade of virtual water, by means of the isolation of two-way exchanges of the same products between importers and exporters (“swap”).

The Italian case, in this sense, is very representative since Italy is a strong importer of virtual water. By using the concept of swap it is highlighted that, even for products which are abundant in Italy, a substantially inefficient trade from the point of view of the budget of virtual water is practiced with increasing intensity. The swap occurs for the economic benefit of the country in question, but raises doubts about its sustainability from the point of view of global management of water resources, in particular with reference to the growing trend in these exchanges.

The considerations expressed in this paper do not contemplate the shipping costs related to the traded goods nor any aspect related to the organoleptic quality of the imported and exported food.

The extension of this work on a larger scale could allow interesting developments in the assessment of the impact of swap and its economic drivers on a global scale, for example with reference to sudden food shortages, deriving both from climate anomalies or from other human interventions. Future activities in this direction will allow us to evaluate the possible relations between virtuous (or vicious) trade policies and country (or global) resilience to droughts, famines etc.

## Supporting Information

Figure S1 **Inefficiency per CROP product in terms of volume of virtual water traded through swap amounts (in 10× units).** Synthetic representations of the virtual water carried from unnecessary imports, per commodity per year, for year 2009. A logarithmic x axis is used to cover the whole range of trading amounts. The category represented is ‘crop’.
(TIF)

Figure S2 **Inefficiency per LIVESTOCK product in terms of volume of virtual water traded through swap amounts (in 10× units).** Synthetic representations of the virtual water carried from unnecessary imports, per commodity per year, for year 2009. A logarithmic x axis is used to cover the whole range of trading amounts. The category represented is ‘livestock’.
(TIF)

Table S1 **Complete list of items considered in the computation of the Virtual Water Volume Ratio.** Of the 162 commodities for which the calculation was possible, 93 present a Virtual Water Volume Ratio higher than 1.
(DOC)

Table S2 **Complete list of commodities for which Italy is conducting a swap, with the referring Value Ratio.** Of the 162 products analysed the calculated Value Ratio is higher than 1 in 123 cases.
(DOC)
