# Supplementary material for: Virtuous and Vicious Virtual Water Trade with Application to Italy
Source: PLoS One. 2014 Apr 11;9(4):e93084. doi: 10.1371/journal.pone.0093084 (PMC3984114; doi:10.1371/journal.pone.0093084)
Supplement: Table S2 — Complete list of commodities for which Italy is conducting a swap, with the referring Value Ratio. Of the 162 products analysed the calculated Value Ratio is higher than 1 in 123 cases. (DOC) [file pone.0093084.s004.doc]

**Table of the Virtual Water Volume Ratio for all commodities**

| **FAO code** | **Item** | **Virtual Water Volume Ratio** |
| --- | --- | --- |
| 1069 | Duck meat | 119.89 |
| 1073 | Goose and guinea fowl meat | 72.69 |
| 907 | Processed Cheese | 57.38 |
| 289 | Sesame seed | 27.57 |
| 290 | Sesame oil | 8.93 |
| 604 | Fruit Tropical Dried Nes | 8.31 |
| 619 | Fruit Fresh Nes | 6.28 |
| 773 | Flax fibre and tow | 5.76 |
| 826 | Tobacco. unmanufactured | 4.77 |
| 882 | Cow milk. whole. fresh | 4.01 |
| 977 | Sheep meat | 3.83 |
| 984 | Cheese of Sheep Milk | 3.56 |
| 558 | Berries Nes | 3.43 |
| 513 | Citrus juice. single strength | 2.84 |
| 515 | Apples | 2.82 |
| 518 | Apple juice. single strength | 2.77 |
| 232 | Walnuts Shelled | 2.75 |
| 222 | Walnuts. with shell | 2.75 |
| 526 | Apricots | 2.74 |
| 1232 | Food Prep Nes | 2.51 |
| 83 | Sorghum | 2.51 |
| 1017 | Goat meat | 2.46 |
| 566 | Marc of Grapes | 2.41 |
| 564 | Wine | 2.29 |
| 239 | Soya Sauce | 2.17 |
| 187 | Peas. dry | 2.1 |
| 461 | Carobs | 2.05 |
| 28 | Rice Husked | 2.03 |
| 238 | Cake of Soybeans | 2.02 |
| 897 | Milk Whole Dried | 2 |
| 521 | Pears | 2 |
| 32 | Rice Broken | 1.99 |
| 563 | Must of Grapes | 1.95 |
| 261 | Olive oil. virgin | 1.94 |
| 1219 | Hair Coarse Nes | 1.92 |
| 237 | Soybean oil | 1.86 |
| 176 | Beans. dry | 1.85 |
| 547 | Raspberries | 1.8 |
| 391 | Paste of Tomatoes | 1.79 |
| 236 | Soybeans | 1.77 |
| 536 | Plums and sloes | 1.69 |
| 560 | Grapes | 1.68 |
| 537 | Plums Dried (Prunes) | 1.66 |
| 108 | Cereals. nes | 1.64 |
| 221 | Almonds. with shell | 1.62 |
| 367 | Asparagus | 1.62 |
| 406 | Garlic | 1.62 |
| 235 | Prepared Nuts (Exc.Groundnuts) | 1.6 |
| 567 | Watermelons | 1.6 |
| 27 | Rice. paddy | 1.54 |
| 220 | Chestnuts | 1.53 |
| 31 | Rice Milled | 1.53 |
| 1034 | Skinsdry Slt Goat | 1.5 |
| 534 | Peaches and nectarines | 1.48 |
| 269 | Sunflower Cake | 1.47 |
| 231 | Almonds Shelled | 1.46 |
| 622 | Fruit Juice Nes | 1.4 |
| 527 | Dry Apricots | 1.39 |
| 544 | Strawberries | 1.37 |
| 191 | Chick peas | 1.36 |
| 517 | Cider Etc | 1.36 |
| 262 | Olives Preserved | 1.36 |
| 471 | Vegetables in Vinegar | 1.36 |
| 268 | Sunflower oil | 1.35 |
| 561 | Raisins | 1.33 |
| 1080 | Turkey meat | 1.31 |
| 562 | Grape Juice | 1.3 |
| 512 | Citrus fruit. nes | 1.29 |
| 60 | Maize oil | 1.29 |
| 890 | Whey Condensed | 1.26 |
| 272 | Cake of Rapeseed | 1.25 |
| 565 | Vermouths&Similar | 1.25 |
| 366 | Artichokes | 1.24 |
| 1037 | Fat of Pigs | 1.23 |
| 267 | Sunflower seed | 1.22 |
| 495 | Tangerines. mandarins. clem. | 1.2 |
| 531 | Cherries | 1.19 |
| 393 | Cauliflowers and broccoli | 1.18 |
| 490 | Oranges | 1.14 |
| 58 | Flour of Maize | 1.13 |
| 426 | Carrots and turnips | 1.11 |
| 260 | Olives | 1.1 |
| 568 | Other melons (inc.cantaloupes) | 1.09 |
| 460 | Veg.Prod.Fresh Or Dried | 1.09 |
| 333 | Linseed | 1.08 |
| 414 | Beans. green | 1.07 |
| 399 | Eggplants (aubergines) | 1.07 |
| 233 | Hazelnuts Shelled | 1.07 |
| 497 | Lemons and limes | 1.02 |
| 56 | Maize | 1.02 |
| 889 | Milk Whole Cond | 1.02 |
| 234 | Nuts. nes | 1.02 |
| 373 | Spinach | 1.01 |
| 417 | Peas. green | 0.99 |
| 270 | Rapeseed | 0.99 |
| 999 | Skins With Wool Sheep | 0.99 |
| 472 | Vegetables Preserved Nes | 0.99 |
| 774 | Flax Tow Waste | 0.97 |
| 463 | Vegetables fresh nes | 0.97 |
| 212 | Flour of Pulses | 0.94 |
| 390 | Juice of Tomatoes | 0.93 |
| 18 | Macaroni | 0.93 |
| 469 | Vegetables Dehydrated | 0.93 |
| 507 | Grapefruit (inc. pomelos) | 0.92 |
| 15 | Wheat | 0.92 |
| 901 | Cheese of Whole Cow Milk | 0.9 |
| 569 | Figs | 0.9 |
| 486 | Bananas | 0.89 |
| 44 | Barley | 0.88 |
| 392 | Tomato Peeled | 0.87 |
| 51 | Beer of Barley | 0.85 |
| 1035 | Pig meat | 0.85 |
| 898 | Milk Skimmed Dry | 0.84 |
| 978 | Offals of Sheep.Edible | 0.83 |
| 116 | Potatoes | 0.83 |
| 1042 | Prep of Pig Meat | 0.82 |
| 1039 | Bacon and Ham | 0.81 |
| 48 | Barley Flour and Grits | 0.81 |
| 46 | Barley Pearled | 0.81 |
| 920 | Hides Wet Salted Cattle | 0.81 |
| 1097 | Horse meat | 0.81 |
| 888 | Milk Skm of Cows | 0.81 |
| 394 | Pumpkins. squash and gourds | 0.81 |
| 886 | Butter Cow Milk | 0.8 |
| 201 | Lentils | 0.8 |
| 909 | Prod.of Nat.Milk Constit | 0.8 |
| 181 | Broad beans. horse beans. dry | 0.79 |
| 872 | Meat of Beef.Drd. Sltd.Smkd | 0.79 |
| 870 | Meat-CattleBoneless(Beef&Veal) | 0.79 |
| 49 | Malt | 0.78 |
| 473 | Vegetable Frozen | 0.78 |
| 921 | Hidesdry S.Cattle | 0.77 |
| 893 | Butterm..Curdl.Acid.Milk | 0.75 |
| 867 | Cattle meat | 0.75 |
| 530 | Sour cherries | 0.75 |
| 1041 | Sausages of Pig Meat | 0.74 |
| 75 | Oats | 0.72 |
| 403 | Onions. dry | 0.72 |
| 372 | Lettuce and chicory | 0.68 |
| 868 | Offals of Cattle. Edible | 0.68 |
| 592 | Kiwi fruit | 0.65 |
| 16 | Flour of Wheat | 0.63 |
| 118 | Frozen Potatoes | 0.59 |
| 334 | Linseed oil | 0.58 |
| 552 | Blueberries | 0.57 |
| 20 | Bread | 0.57 |
| 225 | Hazelnuts. with shell | 0.57 |
| 271 | Rapeseed oil | 0.55 |
| 117 | Potatoes Flour | 0.54 |
| 358 | Cabbages and other brassicas | 0.52 |
| 397 | Cucumbers and gherkins | 0.52 |
| 550 | Currants | 0.52 |
| 72 | Flour of Rye | 0.51 |
| 401 | Chillies and peppers. green | 0.47 |
| 38 | Rice Flour | 0.43 |
| 388 | Tomatoes | 0.43 |
| 76 | Oats Rolled | 0.35 |
| 509 | Juice of Grapefruit | 0.33 |
| 71 | Rye | 0.31 |
| 491 | Orange juice. single strength | 0.24 |
| 122 | Sweet potatoes | 0.24 |
| 223 | Pistachios | 0.19 |
